# Translanguaging as a social justice strategy: the case of teaching Chinese to ethnic minority students in Hong Kong

**DOI:** 10.1007/s12564-022-09795-0

**Published:** 2022-09-25

**Authors:** Danping Wang

**Affiliations:** School of Cultures, Languages and Linguistics, University of Auckland, Auckland, New Zealand

**Keywords:** Translanguaging, Social justice, Ethnic minority, Chinese language, Decoloniality

## Abstract

For the past two decades, a significant number of ethnic minority students from diverse racial, cultural, linguistic, and religious backgrounds have entered Chinese language classrooms in Hong Kong for the first time. Simultaneously, Chinese language teachers have come under criticism for their lack of understanding of diversity and their failure to integrate ethnic minority students academically and socially. However, there is little research on how these teachers can transform their educational beliefs, teaching techniques, and attitudes toward diversity and inclusion to respond effectively to the drastic changes taking place in their professional work. This study examines how a group of Chinese language teachers employed translanguaging as a social justice strategy to address the challenges of teaching minority students in a monolingual and assimilative educational setting in Hong Kong. Classroom observations show that teachers enacted a translanguaging stance, using students’ familiar semiotic resources to make their teaching more inclusive and equitable for ethnic minority students from low socioeconomic and religious backgrounds. Teachers reported becoming more aware of diversity in the classroom as well as of the social inequalities and racial discrimination outside of school. The study shows that criticism has been unfairly levied on Chinese language teachers in Hong Kong, who should not be held responsible for the social problems hindering ethnic minorities’ social mobility. Research should include a decolonial perspective to legitimize translanguaging as a social justice strategy for more transformative praxis in the education sector in postcolonial Hong Kong.

## Introduction

The recent influx of ethnic minority students from different racial, cultural, linguistic, and religious backgrounds into Hong Kong’s mainstream education system has created new challenges for both the education system and the teachers in the classrooms (Gu et al., 2017; Gube & Phillipson, 2019; Loh et al., 2019). Hong Kong has been a Chinese society dominated by homogenous populations throughout its history. The most recent 2016 Population By-Census data (Hong Kong Census & Statistics Department, 2017) show that 92% of Hong Kong residents are ethnic Chinese. The ethnic minority population in Hong Kong numbered 584,383 in 2016, or 8% of its total population, representing an increase of about 70% over 2006. Among all the ethnic minority groups in the territory, the fastest growing is South Asian, including Indians, Nepalese, Pakistanis, and Sri Lankans. Furthermore, the 2016 Population By-Census report (Hong Kong Census & Statistics Department, 2017) reveals that members of ethnic minority communities who were born in Hong Kong increased by about 120% in the decade from 2006 to 2016, from 38,042 to 81,964, respectively. This indicates that more ethnic minorities have settled in Hong Kong, with their children being born and raised locally. The accelerating rate with which ethnic minority children have entered all levels of schooling in Hong Kong has unveiled many historical and deep-seated educational inequalities in Hong Kong’s educational system (Bhowmik, 2017; Loper, 2004).

One of the most crucial challenges faced by ethnic minority students is the language barrier. In Hong Kong, all ethnic minority students are expected to learn Cantonese, the local vernacular, as the spoken Chinese language, as well as traditional Chinese orthography as the written Chinese language (Li & Chuk, 2015), although Mandarin and simplified Chinese are taught in most parts of the world to non-Chinese learners. Apart from the unique linguistic difficulties, Hong Kong lacks a second-language policy for students with different native languages to learn the official language of the territory and perform in academic environments (Loh et al., 2019; Wang, 2019, 2021). Most of Hong Kong’s ethnic minorities, especially South Asians in secondary schools, are expected to study in a monolingual and assimilative educational context to expedite their Chinese learning process and preparation for the university entrance examination (Gu et al., 2019; Shum et al., 2011). Many policymakers have observed that an immersive environment has not been conducive to ethnic minority students quickly acquiring Cantonese as a second language and learning to speak at a native level. Their conclusion was that poor Chinese teaching was to blame (Wang, 2019). In the past decade or so, Chinese language education, its pedagogy, assessment methods, and teachers have been frequently criticized by researchers and the news media for the academic failures of Hong Kong’s ethnic minority students (Loh & Tam, 2016; Shum et al., 2011; Tsung & Gao, 2012; Tsung & Lau, 2019). Bhowmik and Kennedy (2017) found that it has been “common to attribute school failure for ethnic minority students in Hong Kong to problems with Chinese language education” (p. 69). They concluded, however, that there were various reasons why South Asian students did not do well in school, with the ability to speak Chinese being just one of many.

The rapid growth of minority students in native Chinese language classrooms has brought multilevel challenges to Chinese language teachers (Gao, 2012b; Shum et al., 2016). Wang and Tsung (2022) conducted a systematic review of empirical research on Chinese language teaching to ethnic minority students in Hong Kong. They found that existing studies have examined how Chinese language teachers in Hong Kong lack an understanding of diversity and the professional competencies to teach the Chinese language to ethnic minority students. According to these studies, the inability of Chinese language teachers to make minority students proficient in Chinese was often considered the *problem*. Other studies have found that ethnic minority students are taught with the same language acquisition methods used for native speakers (Ng et al., 2020; Shum et al., 2016). Another group of studies found that Chinese language teachers are impolite to or impatient with South Asian students, often ignoring their learning needs and giving them harsher punishments (Bhowmik & Kennedy, 2017). Over the years, the spotlight has been on Chinese language teachers for not being good at their jobs and failing to help ethnic minority students learn Chinese, perform better in school, and fit into society.

However, there have been few acknowledgments that most in-service Chinese language teachers are not professionally trained to teach non-Chinese-speaking students from diverse cultural backgrounds or to help them pass standardized assessments and succeed in university admissions. The 5 years of professional training received by language teachers have played a central role in shaping their professional identity and teaching skills as native language teachers. As Chinese language teachers, they believe their main goal is to educate their students “by passing on Chinese culture and Confucian values” (Gao, 2012a, p. 97). Over the past decade, many Chinese language teachers have made tremendous efforts to upskill themselves with new theories and strategies for teaching ethnic minority students or even to transform their educational beliefs and teaching techniques to ensure that ethnic minority students have equal opportunities as their Chinese peers (Gu et al., 2019). However, little has been heard about teachers’ transformative praxis as they interact with minority students in the classroom. The purpose of this study is to explore how teachers transform their professional practices to cope with the challenges of teaching students from minority groups whose first language is not Chinese in mainstream schools.

## A social justice perspective of language education

The concept of social justice emphasizes distributing wealth, opportunities, and privileges fairly across society, with a special focus on advocating for access to justice for the racialized, marginalized, and minoritized (Truong et al., 2014). Although social justice has been widely used as a guiding principle in health care, law, and social movements in Western countries, it remains an underexplored concept in Asian language education in the Asia–Pacific context.

In education research, the concept of social justice has been often used as a critical lens for understanding the deep issues of equity and justice in teaching underprivileged ethnic minority students (e.g., García et al., 2008; Panagiotopoulou et al., 2020). In recent years, Western educational institutions have started to include official statements on their websites to demonstrate their commitment to equitable opportunities and resources for students from all backgrounds and communities. Focusing on migrants, refugees, and asylum-seekers learning second languages in their adopted countries, language research scholars have warned that the widening social inequalities limit the learning opportunities and social mobility of students from underprivileged backgrounds with different mother tongues (García, 2020; Hurst & Mona, 2017). English language education providers in English-speaking countries are advised to develop equity-minded and inclusive instruction to ensure that all students participate equally in various learning and assessment conditions (García et al., 2008, 2017). However, concepts related to social justice, such as diversity, equity, and inclusion (DEI), were unknown to Chinese language teachers and the Hong Kong education system until the Covid-19 pandemic broke out.

One of the earliest studies that specifically draws on social justice as a conceptual framework is that by Wang and East (2020). Based in New Zealand, they called for Chinese language education providers to be more aware of DEI issues in their Chinese language teaching programs, particularly in terms of making their programs more equitable and accessible during emergency remote teaching. Specifically, they suggested that teaching and assessment design should consider students’ socioeconomic situations rather than assume all students study in a well-resourced environment. A year later, in the United States, Tao et al. (2021) recommended that Chinese language education follow international trends in promoting DEI principles in teaching and teacher training. They called for more professional development to deepen teachers’ understanding of DEI to address diversity in the classroom more fairly. In a recently published book review, inspired by the European language education sector’s commitment to DEI initiatives, Wang and Diao (2021) advocated promoting social justice in the classroom with students from diverse backgrounds. They were particularly concerned with introducing the DEI concepts into Chinese language teacher education to achieve a more profound transformation in teaching Chinese to second-language learners. All the above studies have shown the importance of adopting new paradigms to address issues of diversity and equity in teaching and learning.

In Hong Kong, the deep-rooted racism against ethnic minorities from low socioeconomic backgrounds has created long-term racial segregation both institutionally and socially (Bhowmik & Kennedy, 2017; Gao & Lai, 2018; Lam et al., 2019). Like many other parts of the world, Hong Kong has seen widening socioeconomic gaps and rising educational inequalities in recent decades. Zhou et al. (2016) found that Hong Kong has the highest level of social segregation in secondary schools compared with other Chinese areas such as Macau, Shanghai, and Taipei. Students from low socioeconomic backgrounds are often deprived of opportunities to interact with peers from upper-class backgrounds. Within low-banding schools, underprivileged South Asian students were often isolated by their Chinese peers. Shum et al. (2011) included this quote from a South Asian student: “Chinese students never spoke to us, and we never spoke to them. It was like an invisible separation.” (p. 290). Due to racial segregation, ethnic minorities’ exposure to the Chinese language is extremely limited, even though they live in a predominantly Chinese-speaking society.

The educational inequalities long suffered by South Asians in Hong Kong are well documented. For example, Gao (2019) found that ethnic minorities from poorer backgrounds “are less likely to access, persist, and complete university than their Chinese peers, and are thus locked into the lower socioeconomic strata and are persistently overrepresented in poverty” (p. 136). Thapa and Adamson (2018) found that South Asian students face inequities in schools due to the mainstreaming of either English or Chinese and a lack of opportunities to maintain their own heritage language and identity. The drop-out rates of ethnic minority children from secondary education in Hong Kong have been astonishingly high. Lai et al. (2020) found that social media played a crucial role in disseminating misrepresentations of South Asian peoples and cultures, resulting in prejudice and stereotypical images of the diverse student body both inside and outside of mainstream education. It is impossible to make Hong Kong a fair and just society without addressing the long-term deeply rooted social inequality and racial discrimination against ethnic minorities caused by 155 years of colonialism.

The earliest settlement of South Asians to Hong Kong can be traced back to the expansion of the British Empire in the Asia–Pacific region in the early 1800s (Erni & Leung, 2014). The opium trade led to war between Britain and China, which resulted in the Qing Empire ceding Hong Kong to the British colonizers. Many South Asians were recruited by the British Government to fight for the British Army, and the first major contingent arrived in 1841 to guard the Hong Kong colony as a result of the Opium War (1839–1842). According to Weiss (1991), during the Opium War, it was “defence and security services that the British either did not desire or could not find Chinese recruits, and they sought to meet this need by bringing in Indians” (later Pakistanis and Nepalis) (p. 426). According to Erni and Leung (2014), South Asians remained stationed in Hong Kong until 1997, when British rule ended. In addition to military service, other reasons for South Asian settlements in the early history of Hong Kong included labor requirements, business, and the need for clerks in the colonial government. During the 155 years of British colonial rule, there was little sociocultural interaction between South Asians and the local Chinese population, a situation that was attributed to the British colonial policy of racial and cultural segregation.

Bhowmik and Kennedy (2016) asserted that minority students’ school failure was “more than simply a consequence of academic failure” (p. 69) or a lack of Chinese language skills. Many underprivileged South Asians are limited to low-paid service jobs that require them to be proficient in the local vernacular to communicate with most working-class people in Hong Kong (Lai, 2010). As a minoritized group, they are expected to be assimilated into mainstream Chinese society by studying Chinese to achieve a social class upgrade (Loh & Tam, 2016). In contrast, their wealthier white counterparts have the privilege of thriving in Hong Kong as monolingual English speakers who receive elite English-medium education in fee-paying international schools where Cantonese-speaking is discouraged or even disallowed on campus. The unequal educational systems and opportunities clearly show that the colonial legacy is still shamefully evident in Hong Kong.

In teaching migrant students of diverse backgrounds, cultural responsiveness has been legitimized as a socially just approach to validating students’ prior life experiences in learning the language of host countries (Gay, 2010). According to Villegas and Lucas (2002), culturally responsive teachers are socioculturally aware, know about the lives of their students and see themselves as responsible for and capable of bringing about change to make the schools more equitable. They are cognizant of students’ diverse backgrounds and are often proactive in designing instruction that builds on what their students already know while helping them stretch beyond the already familiar. In Finland, for example, Alisaari et al. (2019) investigated 820 Finnish language teachers about their beliefs and strategies in teaching multilingual migrant students and found that most teachers were positive about incorporating a linguistically and culturally responsive teaching method. Finnish teachers believe such a pedagogy helps connect students’ linguistic and cultural knowledge to new academic knowledge being taught in a new language. In the United States, many studies have been conducted over the past two decades (e.g., Aguirre & del Rosario Zavala, 2013; Gunn et al., 2021; Snyder & Fenner, 2021) to help teachers deepen their pedagogical content knowledge to meet the needs of an increasingly culturally and linguistically diverse student population and to highlight social justice as an essential dimension in the culturally responsive teaching approach. As can be seen, previous studies on such an approach have demonstrated that using linguistic and cultural repertoires and students’ preferred learning styles can make the learning experience of students in the classroom more relevant, meaningful, and effective.

Within Hong Kong’s education system, Chinese language teachers are one of the first professional groups to have close contact with ethnic minority students on a daily basis. Over the years, with increased personal and professional contact with South Asian students in classrooms and through more professional development training in DEI, Chinese language teachers have strengthened their awareness of using a linguistically and culturally responsive teaching approach to create a more meaningful learning environment for students from diverse backgrounds. In a study of Chinese preschool teachers in Hong Kong, Ng et al. (2020) found that many have successfully integrated a linguistically and culturally responsive pedagogy into their classes. In particular, they have developed effective methods for incorporating ethnic minority students’ first/home language(s) and cultures as valuable semiotic repertories to improve classroom interactions and student learning.

## Translanguaging as social justice strategy

Translanguaging is a term originally coined by a Welsh scholar, Cen Williams, to refer to a pedagogical strategy adopted by teachers of using two languages for teaching (Lewis et al., 2012). It offers a new perspective on language teaching and learning by viewing students’ semiotic resources as one repertoire rather than by socially constructed boundaries such as L1 or L2 (Li & García, 2022).

Cenoz and Gorter (2021) defined pedagogical translanguaging as “a theoretical and instructional approach that aims at improving language and content competencies in school contexts using resources from the learner’s whole linguistic repertoire” (p. 1). The introduction of translanguaging in applied linguistics research has fundamentally changed the structuralists’ static view of the boundaries of languages (Li, 2018; Li & Lin, 2019) and has challenged the “monoglossic hegemony” (García, 2020) in language teaching. Translanguaging is not only a language ideology but also a linguistic reality that respects multilingual and superdiverse social conditions, taking multilingual practices as the norm, not the exception. Translanguaging takes a multilingual pedagogical stance that accepts all semiotic inventions of teachers and students (Cenoz & Gorter, 2021).

García and Leiva (2014) argued that translanguaging is “a mechanism for social justice, especially when teaching students from language minoritized communities” (p. 200). In pedagogical translanguaging, teachers are enacting a process of social transformation (García & Leiva, 2014), liberating ethnic minority students from the constraints of the monolingual policies and practices driven by colonial-era ideologies, and creating a plurilingual space for all learners to make sense of their own learning experiences. In a world where translanguaging is the norm for theorizing languages, monolingualism will eventually lose its hegemonic status in maintaining hierarchies of named languages and of the peoples who speak these languages. Indeed, such linguistic hegemonies are partially to blame for the inequalities in today’s world. Canagarajah (2022) noted that the notion of translanguaging as an act of social justice had been widely accepted in the Global North, whereas limited research on this pedagogical process has been conducted in other parts of the world.

South Asia is a highly multilingual place in the Asia–Pacific region (Adinolfi et al., 2022). In Hong Kong, according to Fleming (2015), almost all migrant students with South Asian backgrounds can speak at least two languages, many even four, for family or religious purposes, including Bengali, Hindi, Nepali, Panjabi, Tamil, Urdu, Arabic, and Sanskrit. English is the shared language among South Asians. The multilingual reality for South Asian minorities has underscored the importance of recognizing and respecting ethnic minority students’ prior linguistic and cultural knowledge to engage emergent multilingual learners. Speaking on behalf of Latino students in the U.S., García (2009) asserted that minority students should be regarded as emergent bilinguals whose entire linguistic repertoires should be valued (Li & García, 2022). However, in government documents and previous research, ethnic minority students in Hong Kong are referred to as “Non-Chinese Speaking” students for the convenience of distinguishing native and nonnative speakers of Chinese in schools. Scholars taking a social justice perspective have called for rejecting this deficient and pejorative label (Gao et al., 2019; Wang & Diao, 2021). They argue that the term reinforces the dichotomy between students who can and cannot speak Chinese in educational institutions and, as a result, further worsens the sense of otherness of ethnic minority students in the mainstream environment.

Hong Kong’s mainstream curriculum adopts an assimilative educational model, with ethnic minority students studying in Chinese-medium schools as second-language speakers alongside their native Chinese-speaking peers. However, due to the lack of a second language or bilingual education policy in the territory, the teaching of Chinese to ethnic minority students has not been normalized as *second* language teaching. The Hong Kong government (Hong Kong Education Bureau, 2008) demanded that schools “arrange South Asian students in classes with local students and provide them with focused remedial teaching outside lessons to facilitate their immersion into the Chinese language lessons” (p. 19). Previous research has shown that ethnic minority students in mainstream schools were demotivated by the “target language only” policy implemented in the Chinese classrooms (Bhowmik & Kennedy, 2017). Gao and Shum (2010) found that South Asian students had become more anxious and stressed as a result of the overwhelming immersive mandate. Some mainstream schools had to employ bilingual teaching assistants who speak the students’ home language(s) to provide after-school tutorials. As Wang (2021) argued, there has been an unfortunate mismatch between the one-size-fits-all monolingual policy in mainstream learning contexts and the diverse learning needs of ethnic minority students in Hong Kong.

Existing ethnographic research shows that pedagogical translanguaging has become the de facto classroom interaction strategy (e.g., Cenoz & Gorter, 2021; García et al., 2008; Wang, 2020). Even in the most explicitly regulated environment or bilingual immersion programs, teachers often spontaneously enact pedagogical translanguaging, using a wide range of semiotic resources to facilitate student learning (Tian, 2021). Focusing on translanguaging as a co-initiated pedagogy in Hong Kong, Lin and He (2017) showed that teachers and students were willing to “learn from each other’s linguistic/cultural resources… and are learning and expanding their multiple resources for communication” (p. 243). They found that the translanguaging pedagogy has carved out a space for more meaningful and inclusive learning to take place. In the context of teaching Chinese to ethnic minority students, Ng et al. (2020) found that Chinese teachers have shown a greater awareness of students’ diverse linguistic backgrounds and have changed their attitudes toward students who use their first language with their peers in class. They have adopted an asset-based approach and have come to value students’ linguistic and cultural repertoires, using them to enable a transformative change to make their teaching more inclusive, equitable, and effective.

In Hong Kong, focusing on a beginner Chinese language program designed for adult “school leavers” who dropped out of school for minimum wage jobs, Wang (2019) found that Chinese language teachers had actively explored translanguaging approaches to make their teaching more relevant to ethnic minority students. Based on a classroom ethnography, Wang (2019) identified three major categories of pedagogical translanguaging—interpretive translanguaging (teacher-led for explaining knowledge), managerial translanguaging (teacher-led for giving feedback and building rapport), and interactive translanguaging (student-led for multiple learning needs). Additionally, Gu et al. (2019) found that Chinese teachers often hold a pluralistic language ideology regarding language use, recognizing the positive effect of students’ native languages in brokering between heritage communities and mainstream society. These studies have also shown that teachers are open to flexible classroom language policies to help students make sense of their learning through meaningful interactions (e.g., García et al., 2017; Li, 2018).

Drawing on the concepts of social justice and translanguaging, the present study aims to further the discussion of how teachers enact pedagogical translanguaging as a driver of social justice. It has two research questions: (1) How do Chinese language teachers enact translanguaging when teaching ethnic minority students? And (2) Why do teachers enact translanguaging for ethnic minority students in mainstream educational settings?

## The study

The study is constructed following the principle of classroom ethnography. According to Bloome and Beauchemin (2018), classroom ethnography “seeks to make visible the particular social and cultural practices and processes of everyday life in classrooms” (p. 1). Based on the construct of cultural relativity, classroom ethnography seeks to counter the cultural deficit models of students from nondominant communities whose intelligence, culture, and language are often considered deficient (e.g., Gao et al., 2019). The purpose of classroom ethnographies is to reveal structural inequities and provide insights into how classroom participants reflect and challenge the social contexts in which they are embedded. Using classroom ethnography, this study collected classroom discourses and teacher interview data to generate insights into the two research questions.

### Research context

This study was part of a large-scale project conducted over 1.5 years of fieldwork between April 2016 and August 2018 in a postsecondary education institution. The data collection for this project mainly took place in 2017. The project’s purpose was to develop a more holistic understanding of how teachers employ pedagogical translanguaging in promoting social justice in the classroom. The research site has nine campuses, and Chinese language courses were offered to ethnic minority students on four of them. Most students were South Asians from underprivileged backgrounds, with a small number of Thai, Indonesian, Filipino, and other Asian ethnicities. Students at this research site receive vocational education instead of entering universities.

The Chinese language program involved in the focal project was based on a newly developed curriculum for school leavers who had dropped out of school before completing secondary school with the goal of acquiring basic Chinese language skills to enhance their employability. There were 90 students of South Asian backgrounds enrolled in the program. Each class had approximately 10 to 15 students. Except for a few students who could use Cantonese for greetings and counting, all students in the program were absolute beginners in the Chinese language and all students used English as their common language for communication.

### Teacher participants and the researcher

Eleven Chinese language teachers participated in this research study. Participant recruitment was conducted through purposive sampling. Teachers with ethnic minority students in their classes were specifically invited to participate in the research at the focal research site. Eleven teachers volunteered for this project. Eight of the teachers were female, three were male, and their average age was 38. Eight of the research participants had completed all their degrees at a Hong Kong university, whereas three had obtained a postgraduate degree overseas. They had an average teaching experience of 7.5 years at the research site, indicating they are experienced teachers. All of them are ethnic Chinese and speak Cantonese as their mother tongue. Six teachers identified themselves as predominantly Chinese speakers and five as bilingual Chinese and English speakers. English is the only shared language between the teachers and the minority students. Ethical issues, informed consent, and privacy rights were discussed before data collection, with confidentiality assured to all participants.

The researcher was a tertiary Chinese language educator who has experience in curriculum development and teacher education in the field of Chinese as a Second Language teaching and research. In qualitative research, it is recognized that a researcher’s ontological and epistemological beliefs influence the research (Lin, 2015). This study adopted a reflexive approach to better identify the researcher’s positionality in the process of designing, conducting, and writing about the focal project. The primary motivation for the researcher to conduct the project was to understand how teachers cope with classroom diversity in the context of teaching Chinese to South Asian students.

### Data collection

This study employed two major ethnographic tools in data collection. First, classroom observation was used to collect classroom discourse data to understand how pedagogical translanguaging was enacted in the eleven teacher participants’ classes to empower and engage ethnic minority students. Classroom observation also enabled the researcher to gain a more holistic view of naturalistic and dynamic language practices in the classroom. Every teacher’s class was observed 3–5 times during one teaching semester, depending on the teachers’ availability and willingness to have the research team in class. Each lesson lasted about an hour. To protect participant identities, only audio recordings were made. Follow-up questions about their teaching and activity designs during the classroom observations were asked of the teachers immediately after they finished teaching.

Semistructured in-depth interviews were conducted with individual teacher participants. According to Scanlan (2020), in semistructured in-depth interviews, interviewees are encouraged to talk freely about specific predetermined topics to gather in-depth information around the themes of interest and follow-up questions permit gaining greater understanding of any particular matter. Each interview lasted about 1.5 h. All participants preferred to use Chinese for the interviews. The interviews were audio-recorded and transcribed for analysis. The interview questions were developed based on the literature review and contextualized to fit the Hong Kong case (see Appendix 1). The interviews included 20 questions, categorized into five sections. These guiding questions allowed the researcher to elicit valuable information from teacher participants about (1) their educational, training, and teaching backgrounds; (2) their perceptions and rationales for using any teaching strategies to cope with classroom diversity; and (3) their suggestions for professional development that they desired for improving students’ Chinese learning experiences.

### Data analysis

Recordings of classroom interaction and teacher interviews were first transcribed into textual transcripts. Thereafter, data analysis was performed using a thematic approach. First, in analyzing classroom discourse data, the study used Wang (2019)’s methods of categorization of the pedagogical functions of translanguaging in teaching ethnic minority students. The coding is based on the function of the translanguaged sentence in classroom interaction. This study’s classroom transcripts included all three categories of translanguaging. In the findings section, quotations of the teacher-student interactions were provided to illustrate how translanguaging was enacted by teachers in this study. The coding criteria were as follows:

1. Interpretive translanguaging: e.g., L13 “So 姓 is the last name, 名 is the first name.”
2. Managerial translanguaging: e.g., L11 “好問題 (Good question). Good question.”
3. Interactive translanguaging: e.g., L9 “It is actually the first name, isn’t it?”

The interview data were analyzed to gain insight into the teachers’ motivations for using translanguaging to support ethnic minority students. The thematic analysis emphasizes identifying, analyzing, and interpreting patterns of meaning within qualitative data to understand experiences, thoughts, and behaviors (Kiger & Varpio, 2020). A second coder reviewed the content to ensure the validity of the data analysis and to verify that it was performed in a rigorous manner to yield meaningful and useful results (Nowell et al., 2017). Thematic codes emerged based on repeated words, phrases, and statements that the participants often used when answering the interview questions. Two main categories were found and reported in the second part of the findings section: teachers’ awareness of diversity in the classroom and their awareness of inequalities outside the classroom. Within the classroom, three subcategories were found to be teachers’ primary reasons for using translanguaging: (1) to engage students by incorporating meaningful linguistic and cultural repertoires; (2) to improve the interaction and participation of ethnic minority students in the classroom; and (3) to ensure students receive classroom instruction and information equally.

## Findings

### How do teachers enact translanguaging?

All teachers’ classroom discourses have shown a distinct translanguaging reality. Even though none of the teachers had ever heard of or were familiar with the term translanguaging prior to their participation in the research project, their classroom discursive practices exhibited a “translanguaging reality” (Wang, 2020), in which Cantonese, Mandarin, English, and students’ home languages were all purposefully drawn on for teaching and learning. Teachers spontaneously developed a translanguaging pedagogy to explain the linguistic knowledge of the target language, such as pronunciation, grammar, vocabulary, and cultural concepts, and organized classroom activities to engage all students to actively participate in classroom learning. The classroom discourse is exemplified as follows.

Excerpt 1 (A snapshot of classroom discourse)

**Table:** 

T:	L1	How to pronounce this word?
Ss:	L2	報名 (to sign up, to apply)
T:	L3 L4 L5	Very good 報名 記唔記得 (Do you remember) this character?
S1:	L6	Name?
T:	L7 L8	Yes, name So 報名 is to fill in a form or do something to apply
S2:	L9 L10	It is actually the first name, isn’t it? We’ve got 姓 (surname) from last week as a surname
T:	L11 L12 L13 L14	好問題 (Good question). Good question In Chinese, we have a pair like this—姓名 (name), like this So 姓 (surname) is the last name, 名 (first or full name) is the first name But when you 報名, you need to give your full name, your 姓名 together

The above transcript shows that the classroom conversation data display a clear two-way communication model. Both the teacher and students were active in meaningful negotiations. Regarding the functions of translanguaging, it is not difficult to see that the most frequently used technique was “interpretive translanguaging,” in which new words, grammar, and knowledge-based concepts were explained (e.g., L12, L13, L14). The second group focuses on “managerial translanguaging,” with which teachers give feedback and build relationships with students (e.g., L5, L11). The last group was “interactive translanguaging,” initiated by students for clarification or verifying the meaning of words (e.g., L9, L10).

The study found that teachers took a translanguaging stance in maintaining an interactive classroom discourse to ensure that all students were able to understand their instructional language. Although not all the classroom recordings showed such two-way communication, teachers’ language practices were similar to L12–L14. The recordings show clearly that teachers have developed a greater sense of awareness of student diversity in proficiency levels. They spontaneously initiated culturally responsive teaching methods to make students feel the learning environment was respectful and the learning content relatable. This study has shown that translanguaging is used as a social justice strategy to create an equitable learning environment for students of South Asian backgrounds. The second research question concerns teachers’ increased awareness of their roles in addressing diversity and educational inequalities.

### Why do teachers enact translanguaging?

#### Awareness of diversity in the classroom

All teachers acknowledged that they have moved beyond a state of cultural blindness to one of cultural responsiveness since they began teaching Chinese to ethnic minority students. In the interviews, most found that their experiences of teaching ethnic minority students greatly improved their understanding of diversity in the classroom, pushed them to initiate transformative teaching, attend more professional training opportunities, and develop new teaching techniques to engage their students. Based on the shared agreement that teachers should maximize classroom input from all students, teachers provided three major rationales for enacting translanguaging in teaching.

The first rationale was to engage students by incorporating meaningful linguistic and cultural repertoires of ethnic minority students. For example, T5 introduced a set of new Chinese lexicons to students with religious backgrounds—words that many Chinese people do not use daily and are not normally included in mainstream textbooks. T5 also mentioned that she would prefer to use the English equivalent for these words with students whose Chinese proficiency remained limited.

Excerpt 2 (Interview with T5)

Our textbook is not designed for them, so I included “mosque,” “pray,” “fast,” and “halal food,” words that we (most Chinese) do not frequently use. For beginners, I will speak these words in English, otherwise, there are too many new words for them.

The second reason for teachers to enact translanguaging was to improve ethnic minority students’ classroom participation and better engage them in learning activities. T4 said he realized that using the languages that students were familiar with could get their attention in class, so he learned a few words to speak with his students. He explained that,

Excerpt 3 (Interview with T4)

Speaking their home languages can help get their attention…. So, I learned a little bit of Urdu with my former students. My pronunciation is poor, but it helps me get my students’ attention in class.… They laughed at me.

T4 showed a remarkable professional transformation in his professional development. This finding is inconsistent with Gu et al. (2019), who found in their study that teachers were self-distancing from learning and using students’ home languages in school contexts. Most teachers felt anxious about learning and using an unfamiliar language. Another example was provided by T10, who used students’ cultural knowledge to motivate them to participate in learning activities.

Excerpt 4 (Interview with T10)

I used photos of Indian festivals such as Diwali as visual stimuli for students to engage in group work. So, when they do presentations, I told them they could use Hindi or English for things that do not have an equivalent in Chinese.

The third reason identified in this study was to ensure that students with different Chinese proficiency levels can receive instruction and information equally. T1 explained that her purpose in employing the translanguaging pedagogy was to ensure ethnic minority students were able to follow her instructions in class.

Excerpt 5 (Interview with T1)

I don’t want them to be left out in my interaction with other Chinese students…. When I look in their eyes, they are so lost, and I feel sorry for them…. I was embarrassed to speak English in class because my English pronunciation was not very good. But I know protecting my own feelings will not help my students.

As can be seen, teachers not only used translanguaging for cognitive and pedagogical scaffolding but also for nurturing translanguaging spaces to support students’ emotions and sense of belonging in class. They were aware that minority students felt disconnected due to language barriers, so they developed a translanguaging strategy to make ethnic minority students feel that their learning needs were being attended to.

Teachers’ interviews showed that they became more aware of student diversity and were willing to go the extra mile to achieve a transformation in their teaching. Teachers were willing to step outside their comfort zones to help students stay engaged, empowered, and informed. Teachers said that going to regular professional training sessions, whether they were required or not, helped them learn more about the home cultures and religions of the ethnic minority students.

Excerpt 6 (Interview with T7)

To be honest, I had never felt there was a need to learn about other cultures for doing this job… But since I’ve been teaching South Asian students, I’ve realized how ignorant I was about their cultures and lives.

Teachers found it important to attend professional development programs to learn more about Hong Kong’s ethnic minorities, including their languages, cultures, religions, and the dos and don’ts of embedding their cultures into classroom teaching. They believed that the most important experience they had was through interacting with their students and maintaining respectful relationships with them in the classroom.

#### Awareness of social inequalities outside the classroom

It has become increasingly clear to Chinese language teachers that criticism of Chinese language education in Hong Kong originates from deep-rooted inequalities outside the classroom. With more professional training and closer contact with their ethnic minority students, the teachers revealed that they needed to revisit their preconceptions about marginalized student groups. During the interviews, teachers revealed that they used to describe ethnic minority students as lazy, disobedient, or naughty compared to ethnic Chinese students who attached great importance to Confucian values such as hard work and respect for teachers. One teacher reflected on her past teaching experiences and explained how she developed a greater awareness of social justice toward ethnic minority students in her Chinese class.

Excerpt 7 (Interview with T6)

When I first started teaching them (in 2006), they did not want to study Chinese… I had avoided teaching them because I found them to be not hard-working and disrespectful. So, if they spoke other languages in my class, I’d punish them.… now I realize it was because they did not feel accepted by their peers. It is about their skin color, not their Chinese language skills.

The teacher pointed out that given the rampant social discrimination against South Asians in the labor market and the media (Lai et al., 2020; Thapa & Adamson, 2018), being able to speak Cantonese would help them “make a favorable impression in front of the Chinese people but may not guarantee their acceptance into mainstream society” (T6). This also corresponds with Wong’s (2020) study. Students’ low opinions about themselves and their Chinese language skills have a negative impact on their motivation to learn Chinese, their academic performance, and their social integration. Teacher interviews confirmed that Chinese language education for ethnic minorities is an issue that goes far beyond teacher incompetency.

Another teacher shared that students’ motivation for learning Chinese has been more exam-driven than integration-focused. Teachers perceived that students were more motivated to learn Chinese in the last decade than in previous generations, despite the fact that their intrinsic interest in the Chinese language and culture remained secondary to that of English. The teacher explained,

Excerpt 8 (Interview with T2)

Many of them are not interested in diving deeper into the Chinese-speaking world. They desire a lifestyle in the CBD, speaking English with bankers and lawyers. All I can do is use a language they understand to win their hearts and minds and show them that Chinese language and culture can be interesting and useful for their future careers.

T2 reported that her students viewed Chinese language learning as no more than a short-term strategy for meeting qualifications and taking examinations, whereas they learn, use, and enjoy English because it is their desired Western way of life.

As can be seen, the critical opinions of T6 and T2 on the unique challenges of teaching ethnic minority students are pertinent to neocolonial thinking and inequalities in the macro sociopolitical and historical context outside the classroom in Hong Kong. In particular, T2’s disappointment in students because they desired to become monolingual English speakers has a strong link with Hong Kong’s colonial legacy, that is, the link between being a monolingual English speaker and high income and authority (Loh et al., 2019).

## Discussion

By examining how Chinese language teachers adopt a translanguaging stance through the lens of social justice, this study considers how ground-level transformative praxis takes place in the everyday language classroom. Based on classroom observation and teacher interviews, the study has highlighted how Chinese language teachers in Hong Kong enact pedagogical translanguaging in their interactions with underprivileged minority students in class and, more significantly, why they no longer adhere to a monoglossic ideology in the assimilative and monolingual environment. The findings demonstrate how Chinese language teachers hold a pluralistic language ideology in teaching ethnic minority students, which corresponds to what Gu et al. (2019) found in Chinese language teachers. The study confirms that Hong Kong’s Chinese language education for ethnic minority students is a unique case, which requires new perspectives to break away from the deficit-based, neocolonial ideologies and epistemologies.

This classroom ethnography demonstrated that in-service teachers had initiated pedagogical translanguaging on their own, through trial and error, to more effectively engage ethnic minority students. Teacher participants demonstrated admirable efforts and substantial agency in overcoming the challenges of coping with classroom changes. Despite the fact that none of the teachers had been trained specifically in using a translanguaging pedagogy or had been exposed to this concept, many developed grassroots translanguaging strategies to make their teaching more inclusive and equitable for their students.

The findings show that Chinese language teachers have selflessly invested their time adapting teaching materials and attending professional development seminars and workshops to create more desirable learning environments for students from diverse backgrounds. Although this study was conducted with a small group of teachers, it has challenged the prevalent perceptions of Chinese language teachers as incapable of handling diversity in the classroom and, more generally, of the monolingual hegemony in Chinese language education (Wang, 2015). More importantly, the teachers had become more conscious of the multilevel social impact on Chinese language teaching in Hong Kong (Bhowmik & Kennedy, 2017), which demanded that they follow the one-size-fits-all monolingual principle and, at the same time, criticized them for not being flexible or open-minded enough to address diversity in the classroom.

Taking a translanguaging perspective, this study suggests that the teaching of the Chinese language to ethnic minorities should adopt a repertoire-based approach (Cenoz & Gorter, 2021). Policies and pedagogies should not be developed to force students to ignore or forget the rich semiotic resources upon which their entire life experiences have been built. Conversely, to achieve more equitable and inclusive educational goals, Chinese language educators must abandon the previous cultural deficit-based approach. Based on the critical review of the literature and the classroom ethnography, this study finds it vital to promote translanguaging as a social justice strategy to better recognize ethnic minority students’ unique linguistic and cultural resources. Integrating DEI topics into language education is vital for improving not only the Chinese learning experiences of ethnic minority students but also their life-long engagement with Chinese society.

The study highlights that the case of Hong Kong’s Chinese language education is a complex sociopolitical issue rather than a pure educational problem that can be “fixed” within the classroom alone. This study has provided valuable data for researchers to understand the teacher-student interactions and teachers’ own analysis of their improved understanding of student diversity and the interplay of macro-, meso-, and microlevel influences on teachers’ language ideologies and adoption of a translanguaging stance. Due to the lack of research on translanguaging (Wang & Tsung, 2022), teachers’ efforts in adapting their teaching approaches and their growing awareness of social inequalities were not valued or legitimized as a progressive mechanism for social justice (García & Leiva, 2014). This is one of the first studies to draw on concepts of social justice and translanguaging to understand the lived experiences of language instructors teaching Chinese to minority students in mainstream educational contexts. It has important implications for Chinese language teaching and language teacher education throughout the Asia–Pacific region.

Finally, it suggests that future research on Chinese language education and policymaking should take a decolonial turn to expose and overcome the legacy of colonialism that is still deeply entrenched in Hong Kong today. The study calls for a critical examination of the root issues that caused and reproduced the inequalities and injustices in our education system and societies founded on the colonial experience. This study’s literature and findings have shown that colonialism’s legacy is still predominant in Hong Kong’s education system and in the attitudes and understandings of researchers (Wang & Tsung, 2022). Hong Kong’s language education must enact a socially just approach to actively engage in enlightened discussions toward a decolonizing approach to teaching minority students. It is time to adopt new theoretical tools in research and policymaking that challenge the cognitive colonialism (Criser & Malakaj, 2020) that still governs our lives and reproduces structural inequalities, discrimination, bias, and racism in language education, especially for minority and marginalized students.

## Conclusion

This study found that a group of in-service Chinese language teachers in a postsecondary institution in Hong Kong developed a spontaneous translanguaging pedagogy using semiotic resources familiar to ethnic minority students to engage students in meaningful content learning, maintain classroom interaction, and ensure students receive classroom instruction equally. Their transformative teaching practices were enacted due to their increased awareness of student diversity in the classroom and social inequalities outside the classroom. Using translanguaging as a social justice strategy, the teacher participants in this study created a more inclusive and equitable learning environment for ethnic minority students.

Specifically, this study provides insights into how translanguaging might prompt pre-and in-service teachers alike to acknowledge the constraints of monolingual language pedagogies (Deroo & Ponzio, 2019) and take a bottom-up approach to explore alternative ways of engaging and empowering minoritized students in mainstream education contexts (García & Leiva, 2014). Translanguaging not only has the potential to challenge inequalities at the systemic and institutional levels, but it can also help teachers push back against monolingual ideologies and adopt more inclusive perspectives of dynamic multilingualism.

This study has several important implications for language teaching and teacher education in the Asia–Pacific region and beyond. First, translanguaging strategies and pedagogies should be formally included in teachers’ professional training to deal with the increasing student diversity in language classrooms worldwide. Li (2018) emphasized that “translanguaging empowers both the learner and the teacher, transforms the power relations, and focuses the process of teaching and learning on making meaning, enhancing experience, and developing identity” (p. 7). By helping students learn, teachers not only enhance their own teaching but also contribute to a better society. Second, social justice and DEI are new agendas that need to be incorporated into the professional standards for teacher certification in Asia–Pacific countries. Integrating a social justice framework into teacher education is essential for promoting equitable and inclusive teaching and is conducive to establishing goals related to intercultural competencies that facilitate learning how to better interact with people from different backgrounds. Finally, it is imperative to adopt a decolonial perspective to legitimize translanguaging as a social justice strategy for more transformative praxis throughout the Asia–Pacific region.

## Appendix 1: Interview questions (native speaker teachers’ ideologies in teaching ethnic minority students)

### Teaching experience

1. What are your degrees and professional backgrounds?
2. What languages or dialects do you speak, and to what levels?
3. How long have you been teaching Chinese to ethnic minority students?
4. What proficiency levels have you taught to ethnic minority students?

### Student backgrounds

5. What are your students’ backgrounds (ethnicity, culture, religion, gender, age, etc.)?
6. What do you think of your students’ motivation to learn Chinese?
7. To your knowledge, what languages do they speak, and to what levels?
8. What do you think of the roles of these languages in their life experience?

### Pedagogical adaptions

9. In what languages do you communicate with them on campus and in your class?
10. Compared to teaching ethnic Chinese students, what are the biggest challenges in teaching ethnic minority students?
11. How do you address these challenges? How different are your approaches from teaching native speakers?

### Perspectives on language policies

12. Does your institute have a medium of instruction policy? Do you have your own?
13. Do you find it helpful to use students’ first or familiar languages in teaching Chinese?
14. If so, why do you do this, and how will it help ethnic minority students?
15. Do you normally allow students to use their first or familiar languages in your class?
16. How do you feel when students speak a language that you don’t know in class?
17. What do you think of the immersion approach or “target language only” approach?
18. Have you attempted a “target language only” approach? Why and why not?

### Suggestions for solutions

19. What professional development activities would help you teach ethnic minority students more effectively?
20. What can the institute and the government do to improve ethnic minority students’ education and social integration?
